# First report of *Neospora caninum* from aborted fetuses of cattle, sheep, and goats in Bangladesh

**DOI:** 10.5455/javar.2024.k811

**Published:** 2024-09-29

**Authors:** Md. Shahiduzzaman, Pijush Biswas, Ajran Kabir, Abu Rakib M. Beni Amin, Sakhyajit Saha Parijat, Nurnabi Ahmed, Md. Zawad Hossain, Majed H. Wakid

**Affiliations:** 1 Department of Parasitology, Faculty of Veterinary Science, Bangladesh Agricultural University, Mymensingh, Bangladesh; 2 Department of Microbiology and Hygiene, Faculty of Veterinary Science, Bangladesh Agricultural University, Mymensingh, Bangladesh; 3 Faculty of Veterinary Science, Bangladesh Agricultural University, Mymensingh, Bangladesh; 4 Department of Medical Laboratory Sciences, Faculty of Applied Medical Sciences, King Abdulaziz University, Jeddah, Saudi Arabia; 5 Special Infectious Agents Unit, King Fahd Medical Research Center, King Abdulaziz University, Jeddah, Saudi Arabia

**Keywords:** Aborted fetuses, cattle, goat, sheep, nested-PCR, Neospora caninum

## Abstract

**Objectives:**

The study aimed to detect *Neospora caninum *by nested PCR (nPCR) in aborted fetuses of cattle, sheep, and goats in Bangladesh.

**Materials and Methods:**

The head portion of each aborted fetus (111) was dissected at each sampling site and transferred to the laboratory in an ice box. Data on risk factors associated with *N. caninum* infection were simultaneously collected. Deoxyribonucleic acid was extracted from brain tissue to perform nPCR targeting the internal transcribed spacer 1 (ITS1) ribosomal DNA (rDNA) gene of *N. caninum *and sequencing was performed from the representative positive samples*. *

**Results:**

By nPCR, *N. caninum* was found in 16.0% of aborted fetuses of cattle, followed by sheep (14.81%) and goats (11.78%). The highest prevalence was found in aborted fetuses of animals during the second trimester (27.78%) of pregnancy aged 2 to 4 years (18.75%). Obtained sequences showed they were completely matched with *N. caninum *ITS1 rDNA gene deposited in GenBank. Univariate analysis demonstrated that pregnancy stages (trimesters), abortion history of the animals, and access to dogs in animal farms were significantly (*p* ≤ 0.05) correlated with *N. caninum *infection.

**Conclusion:**

This study represents the first investigation into the molecular detection, phylogenetic characterization, and analysis of risk factors associated with *N. caninum *in livestock in Bangladesh. According to the research findings, *N. caninum* infection may have a role in abortion cases and the ensuing financial losses in the nation’s livestock industry.

## Introduction

*Neospora caninum* is an intracellular apicomplexan protozoan parasite that is the leading cause of abortion in livestock and responsible for neurological disorders in carnivores [1]**.** This coccidian parasite is distributed worldwide and has a wide host range [2,3]. Dogs and coyotes are the final hosts of *N. caninum*, while the intermediate host includes a wide range of warm-blooded animals such as sheep, cattle, goats, deer, rhinoceros, rodents, and horses [4,5]. In cattle, *N. caninum* is a major cause of abortion worldwide, and calves with congenital infections can show neurological signs [6]. This parasite usually causes abortion at 5–6 months of gestation, but in cattle, it can cause abortion at any stage of pregnancy [4]. In dogs, *N. caninum* can affect the central nervous system, brain, liver, muscle, and other visceral tissues [5]. The major route of infection in ruminants is transplacental or vertical [4]. Postnatal or horizontal transmission is possible through the ingestion of tissues containing cysts and tachyzoites or through the ingestion of sporulated oocysts in contaminated food or drink [7].

Several studies in Bangladesh indicated that abortion in cows is one of the reasons for production loss in dairy farming [7]. A serological study on bulk milk (92) and blood serum (184) samples from cattle of the Chittagong, Satkhira, and Sirajgong districts reported a 1.81% overall prevalence of neosporosis. Among the tested bulk milk samples, 5.43% were found to be seropositive, whereas no serum was found positive [8]. The authors concluded that *N. caninum* is possibly distributed throughout the country.

There is no molecular data available on the prevalence of *N. caninum-*related abortion in animals in Bangladesh, although the case of abortion in ruminants or livestock is significant [9], and frequent abortion occurs in high-yielding dairy farms in Bangladesh (personal communication). Cattle in farms that are in close contact with dogs are at risk for acquiring *Neospora* infection [6]. In Bangladesh, most of the dogs or other canine species are stray (free-roaming) in nature [10]. Since most of the farms are semi-intensive or free-range with lack strict bio-security practices, there is a chance of getting the stray dog into the farm and facilities (observation during our study) as reported [11]. Therefore, contamination and transmission of *N. ­caninum *in livestock can frequently occur from dog feces in Bangladesh.

*Neospora caninum* is not known to naturally infect primates or humans, but serological evidence suggests that humans are exposed to *N. caninum* [12,13], and experimental infections in rhesus macaque tachyzoite crossed the placenta and infected the fetus [13]. Although infection of *N. caninum* is found in several organs, the fetal brain is the most consistently affected organ [14]. Experimental infection in mice with *N. caninum* suggests that damage to neuronal tissues in the frontal lobe, medulla oblongata, and cerebellum is associated with the pathogenesis of neosporosis [15].

The diagnosis of *N. caninum* infection depends on the chosen methods and the state of the affected tissues. Several methods have been used to detect and characterize *N. caninum* infection in aborted fetuses, including histopathology [15], immunohistochemistry (IHC) [16], polymerase chain reaction (PCR) [17], and more sensitive and specific nested PCR (nPCR) [17]. Most aborted fetuses are likely to be autolyzed; it is therefore difficult for histological examination [18]. Only a few *N caninum* may be present in autolyzed tissues that can be sensitively detected by nPCR [18]. Frozen and/or autolyzed brain samples can hamper histopathological and IHC diagnosis because they can destroy the cellular architecture of the parasite [18]. IHC is a specialized method that works well on well-fixed formalin-impermeable tissues and has a modest sensitivity [16]. Formalin fixation of the tissue can also affect the sample’s immunoreactivity because it creates “methylene bridges,” or numerous linkages with proteins that obscure epitopes and prevent antibodies from binding to the antigen. Although the diagnosis of abortion due to *N. caninum* can be sensitively and specifically detected by IHC, the PCR method is both specific and more sensitive [18]. However, molecular techniques can detect deoxyribonucleic acid (DNA) in small quantities of fetal samples, whatever their condition, whether mummified or in varying degrees of autolysis [18]. The ribosomal DNA (rDNA) internal transcribed spacer 1 (ITS1) region is a good marker for the ­distinction of *N. caninum* from other closely related parasites [19].

Given the clinical, epidemiological, and economic significance of abortion in ruminants in Bangladesh, this study aimed to detect *N. caninum* in aborted fetuses of cattle, sheep, and goats using nPCR. Additionally, the study aimed to identify the risk factors associated with this parasitic infection.

## Materials and Methods

### Ethical approval

The study was approved by the Animal Welfare and Experimentation Ethical Committee of Bangladesh Agricultural University. The approval number is AWEEC/BAU/2021(57).

### Study area 

**Figure 1.**
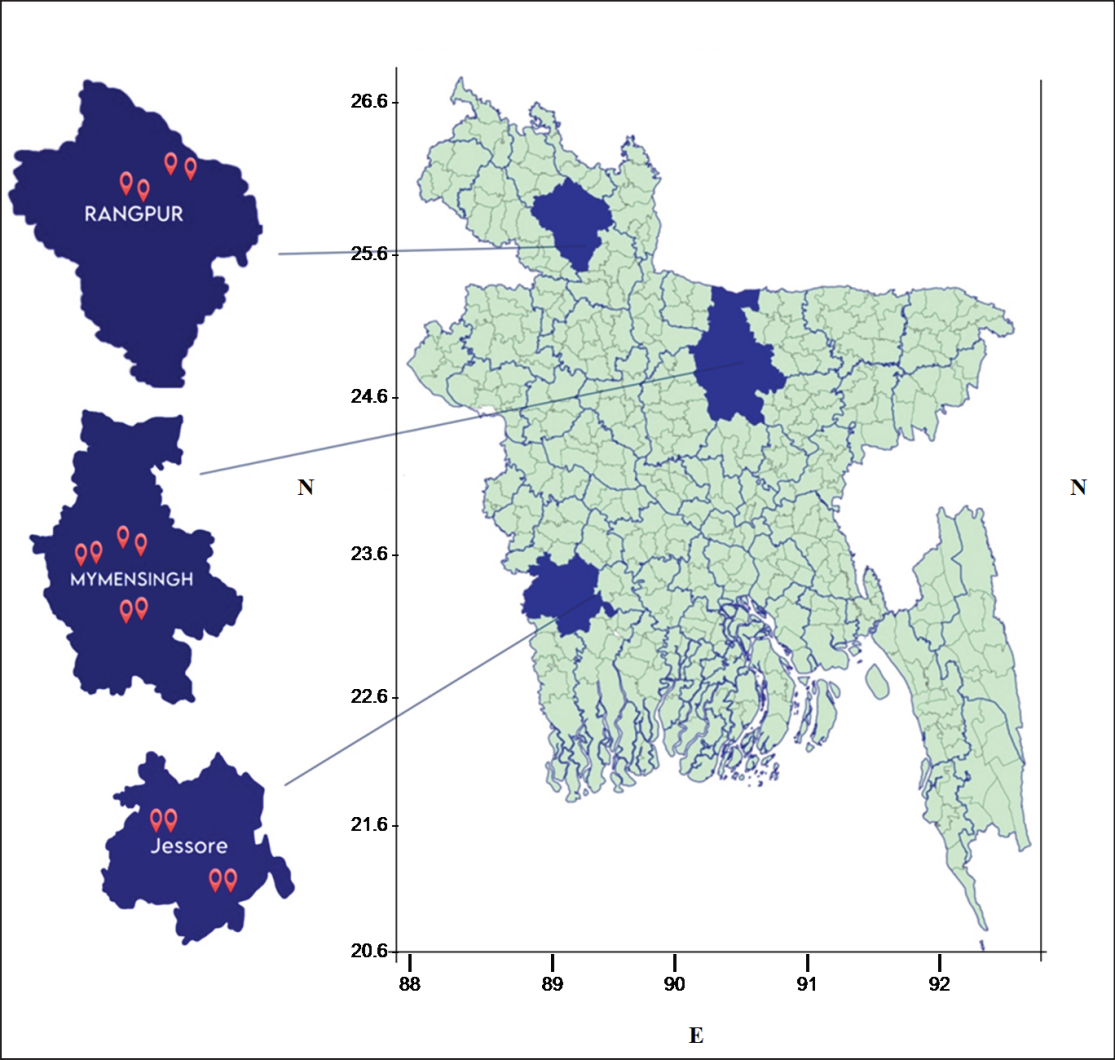
Colored areas of map showing the study areas of Bangladesh. Location symbols in the magnified districts (Jessore, Mymensigh and Rangpur) show the specific location in the study areas.

We selected Jhikargachha and Monirumpur Upazila of Jessore (23.16° N, 89.21° E); Sadar, Trishal, and Fulbaria Upazila of Mymensingh (24.75° N, 90.42° E); and Sadar, and Kaunia Upazila of Rangpur (25.75° N, 89.26° E) districts (divisional units) of Bangladesh (Fig. 1). The reason for selecting these three districts was a recent report of frequent abortions both in farms and household livestock there (personal communication, phone calls, veterinary doctor, and livestock office personnel reports).

### Collection of aborted fetuses and relevant information 

In total, 111 heads of aborted fetuses (50 cattle, 27 sheep, and 34 goats) were collected from 3 different districts (Jessore, Mymensingh, and Rangpur) in Bangladesh (Table 1). The cool chain was maintained during the transportation of the head portion of the aborted fetuses to the laboratory at the Department of Parasitology, Bangladesh Agricultural University, Mymenisngh. The aborted fetuses were collected with the help of veterinarians, farm managers, and persons familiar to us at each site. A closed-ended questionnaire was used to collect information on age, breed of animal, abortion period, previous abortion history, access to dogs on the farm, and management systems.

**Table 1.** Prevalence of *N. caninum* infection in cattle, sheep, and goats in relation to risk factors.

Risk factor	Variables	Total examined	% positive	Odd ratio	Confidence interval 95%	*p*-value
Species	Cattle	50	16.0			
	Sheep	27	14.81	1.095	0.297–4.033	0.891
	Goat	34	11.76	1.304	0.294–5.780	0.727
Breed	Local	70	17.14			0.291
	Cross	41	9.76	1.914	0.574–6.382	
Age	≤ 2 years	23	8.70			
	2–4 years	48	18.75	0.413	0.082–2.088	0.285
	4–6 years	40	15.0	1.615	0.494–5.282	0.428
Locality	Jessore	36	16.67			
	Mymensingh	43	11.63	1.520	0.423–5.466	0.992
	Rangpur	32	15.63	0.711	0.187–2.698	0.616
Abortion period	First trimester	32	3.13			
	Second trimester	54	27.78	0.084	0.011–0.670	0.019
	Third trimester	25	0			
Previous abortion history	No	76	3.95	–	–	0.0001
	Yes	35	37.14	0.070	0.018–0.266	
Presence of dog	No	43	2.33			0.017
	Yes	68	22.06	0.081	0.010–0.635	
Management system	Intensive	17	0			
	Semi-intensive	29	24.14	0.086	0.005–1.605	0.100
	Free range	65	13.86	1.980	0.656–5.972	0.225

### Brain tissue collection and processing 

Since the brain is considered the organ of choice for diagnosis of *N. caninum* in aborted fetuses [20], we excised the brain from each head portion of aborted fetuses. Tissues from the left and right lobes (five different points—front, 2 lateral, and posterior) of the isolated brain were taken in a 2 ml sterilized Eppendorf tube. The brain samples were stored at −20°C until DNA extraction.

### DNA extraction 

For each sample, 50 mg of brain tissue was cut into pieces, homogenized with distilled water, and DNA was extracted according to manufacturer instructions (Wizard Genomic DNA Purification Kit, Promega, USA). A nanodrop (NanoDropTM, Thermo Fisher, USA) was used to test the isolated DNA’s concentration.DNA samples were stored at −20°C until further analysis.

### nPCR and gel electrophoresis

nPCR was used to amplify ~300 bp of *N. caninum* DNA fragment of the ITS1 (internal transcribed spacer) gene [21]. The primer pairs were: NN1 (5′- TCAACCTTTGAATCCCAA -3′), NN2 (5′- CGAGCCAAGACATCCATT -3′), NP1 (5′- TACTACTCCCTGTGAGTTG -3′), and NP2 (5′- TACTACTCCCTGTGAGTTG -3′). The primary reaction was performed in a Mini PCR (Oxford University), with a 25 μl reaction volume consisting of 12.5 μl GoTaq^®^ Green Master mix (Promega, USA), 10 pmol each primer (NN1, NN2), and 3 μl DNA. The initial denaturation for 5 min at a temperature of 95°C, then 35 cycles of 1 min denaturation (95°C), 1 min annealing (55°C), and 1 min extension (72°C), followed by a final 5 min extension (72°C).

The secondary reaction was performed with NP1 and NP2 primers, 2 μl of the primary amplification product under the same primary PCR conditions (except annealing temperature of 53°C). Positive control was used from previously identified *N. caninum* DNA by sequencing and ultrapure water as a negative control. The analysis of PCR products was run by 1.5% agarose gel electrophoresis.

### Sequencing 

Six positive PCR products with clear thick bands demonstrated from nPCR of cattle (2), sheep (2), and goat (2) samples were subjected to sequencing for further confirming *N. caninum*. The nPCR products were purified by the Wizard SV gel and PCR clean-up system (Promega, USA), and the sequencing was performed at DNA Solution Ltd. (Dhaka, Bangladesh) using an ABI 3500 Dx Genetic analyzer (Applied Biosystems, USA).

### Phylogenetic analysis

ITS-1 sequences were aligned with MEGA v.11.0’s Clustal W tool [22]. The sequences were compared with best-hit-scoring ITS1 *N. caninum* DNA sequences deposited in the GenBank database using the National Center for Biotechnology Information Basic Local Alignment Search Tool (BLAST). A neighbor-joining phylogenetic tree of *N. caninum* was constructed in MEGA v.11.0 software using the Tamura-Nei model, and bootstrap values were calculated using 1,000 replicates. *Eimeria brunette *(AF446057.1) was used as an outgroup. Representative sequences found in this investigation were added to the GenBank. With an accession number ranging from QQ398253 to QQ398258, one can obtain the gene sequences from GenBank.

### Data management and analyses

The data were analyzed using IBM Statistical Package for the Social Sciences Statistics for Windows, Version 25.0. (Armonk, NY: IBM Corp.). Chi*-*square was performed to compare the prevalence rates of neosporosis among different animal species, age, breed, abortion trimester, abortion history, access of dogs in the farms, and management practices. Differences were considered significant when *p-*value ≤0.05. Univariate logistic regression was performed to study the effects of risk factors on *N. caninum* infection in animals.

## Results

### Prevalence of N. caninum

**Figure 2.**
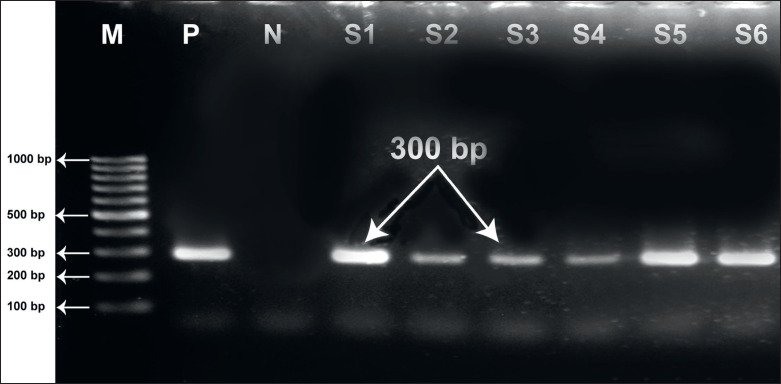
Gel electrophoresis showing nPCR amplicons of the *N. caninum* ITS1 gene fragment (300 bp). In lanes: M, molecular weight marker = 1 kbp; P, positive control; N, negative control; S1–S6 are samples (S1, S2 cattle; S3, S4, sheep; S5, S6 goat samples). The sequence confirmed sample was used as positive control.

The detection of the *N. caninum* ITS1 gene was confirmed by the observation of about 300 bp band in nPCR (Fig. 2). Among the 111 aborted fetuses, only 16 were found positive (14.41%) for *N. caninum* by nPCR. The prevalence of *N. caninum* in cattle was 16.0% (8/50), 14.81% (4/27) in sheep, and 11.76% (4/34) in goats. In Jessore, the prevalence of *N. caninum* was 16.67% (6/36) while 11.63% in Mymensingh (5/43) and 15.63% in Rangpur (5/32) district. However, no significant relationships were observed between *N. caninum* infection and the abortion of animals in this study in relation to species, age, breed, and locality. The highest prevalence (21.51%) was found in animals in which abortion occurred in the second trimester. *Neospora caninum *was considerably more (18.75%) in animals aged 2 to 4 years compared to other age groups (Table 1).

### Phylogenetic analysis

**Figure 3.**
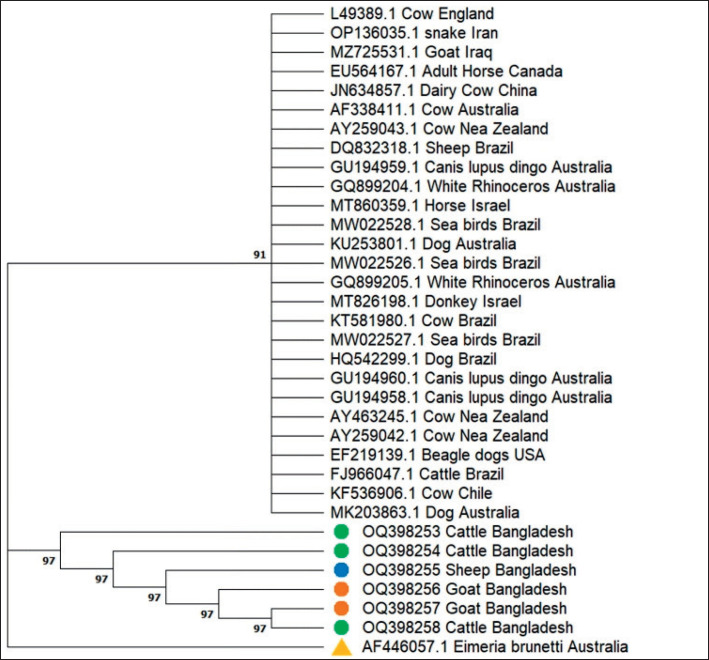
Neighbor-joining phylogeny of ITS1 gene sequences of *N. caninum* species. The percentage at branch points is associated taxa clustered together of 1,000 bootstrap data sets that supported the specific internal branches. The tree is drawn to scale, with branch lengths in the same units as those of the evolutionary distances used to infer the phylogenetic tree. GenBank accession numbers accompany each taxon name. Green circle: *N. caninum* sequences isolated from brain tissue of aborted fetuses of cattle, sheep and goat in Bangladesh. Red triangle: an out-group.

The nucleotide sequences *of N. caninum *for different species discretely made position irrespective of region or study location. The neighbor-joining tree of ITS1 sequences of *N. caninum *demonstrated that *N. caninum *isolates of this study clustered with previously established *N. caninum *sequences with strong nodal support (91% by bootstrapping value). There were very close relationships among the Bangladeshi isolates, with a strong nodal support value of 98% by bootstrapping (Fig. 3). Phylogenetic analysis indicated that *N. caninum *is genetically identical, belongs to different hosts and geographical areas, and is grouped into the *N. caninum*-clade (Fig. 3). BLAST analyses of the ITS1 rDNA gene showed 99%–100% similarities between *N. caninum* sequences deposited in GenBank.

### Risk factors

Among the risk factors studied, abortion in the second trimester of pregnancy (*p* = 0.019), previous abortion history of the animals (*p* = 0.0001), and access to dogs in animal farms (*p* = 0.017) were found to be significant factors (Table 1). Older animals (above 2 years) were more likely to be infected with *N. caninum* compared to younger ones (less than 2 years) (odd ratio 0.413 to 1.615) (Table 1). Likewise, there were higher odds (1.98) of infectivity in free-range animals compared to those raised semi-intensively, as well as in farms with the presence of stray dogs relative to those without dogs (Table 1). Farms with crossbred animals were more likely to be infected with *N. caninum* compared to those with local breeds (odd ratio 1.914). Farms with a previous history of abortion are more likely infected with *N. caninum* than those without a history of abortion. The univariate logistic regression analysis’s findings show that the prevalence of *N. caninum* in the studied areas is not significantly influenced by species, breed, age, location, or management strategy.

## Discussion

Many undiagnosed cases of abortion, stillbirth, and retained placentas significantly affect livestock development in Bangladesh [23]. A number of studies on abortion in sheep, goats, and cattle have been conducted in various countries; however, there is no information available regarding the incidence of *N. caninum* DNA in these producing animals in Bangladesh. To our knowledge, this is the first molecular report and phylogenetic analysis of *N. caninum* in aborted fetuses of cattle, sheep, and goats in Bangladesh.

Several PCR-based methods have been developed, focusing on the 18S rDNA, 28S rDNA Nc5, and the ITS1 region sequence specific for *N. caninum* [24]. Using a particular gene, the nPCR has a high sensitivity and specificity for detecting the parasite [25].

The brain is considered by several authors to be the reference organ for the amplification of the ITS-1 fragment of *N. caninum* [18,20]. Due to the similarity between *N. caninum* and other coccidians (*Toxoplasma gondii *and* Hammondia heydorni*), the sequencing of ITS1 can be applied as a complementary tool in the identification of species and strains of *Neospora* [19]. In our study, *N. caninum*-specific nPCR of the ITS-1 sequence with the primer pairs NN1/NN2 and NP1/NP2 [21] ensured the specificity of the test in the detection of *N. caninum,* and BLAST analysis confirmed that the primers of the ITS1 region of the nPCR were specific to this parasite. Using nPCR with a pair of NN and NP primers, *N. caninum* was found in the brains of 16 (14.41%) of the 111 analyzed aborted fetuses in the current study.

The status of animal infection with *N. caninum *in Bangladesh is unknown. Only a serological study has been performed where positive samples were detected in bulk milk of cows but no serum was found positive, and results were presented in a conference [8]. Although it is important to know the current status of *N. caninum* in different animals, assessing the prevalence of this parasite in cattle, sheep, and goats is imperative, because of its economic and ecological significance as well as for designing the control programs.

Since the transplacental transmission is the main route of *N. caninum infection* among animals, we collected aborted fetuses of animals for detection of the causal agent. Furthermore, we chose brain tissue from the aborted fetuses because the brain is the organ of choice for diagnosis of neosporosis [20].

The overall prevalence rate of *N. caninum* infection in the studied aborted fetuses was 16.0%. The overall prevalence rates of *N. caninum* infection in bovine aborted fetuses globally were 35% and 43% using serological and molecular methods [2]. However, the higher rate of prevalence in our study and other studies might be due to the collection of samples from aborted fetuses rather than serological detection in randomly selected mother animals [26].

The global prevalence of *N. caninum* in aborted fetuses of sheep and goats using molecular methods was reported to be 7%–15% [2,3]. In our study, the prevalence of *N. caninum* was 14.81% in aborted fetuses of sheep and 11.76% in goats, which falls within the range reported in previous studies [1]. The global prevalence of *N. caninum *ranged from 12% to 42% of aborted fetuses from dairy cattle, which further supports our results. However, the observed variation in the global prevalence might be due to the differences in the sample size, geography, and different types of study methods. The meta-analysis conducted by Nayeri et al. [2] revealed that the prevalence of *N. caninum* infection in the bovine aborted fetuses was assessed to be 41%, 50%, and 31% depending on diagnostic methods such as PCR, nPCR, and others. This suggests that nPCR has a higher sensitivity for detecting *N. caninum*.

The prevalence of *N. caninum* infection did not exhibit significant variations among the three distinct study areas (districts). However, these results do indicate that *N. caninum* infection might be widespread across a substantial portion of the country.

In this study, six positive samples (2 from each animal species) were sequenced for phylogenetic analysis. The sequences described in this study shared higher homology (99%–100%) and the same gene (ITS1 region) sequences of *N. caninum* deposited in GenBank. Phylogenetic research revealed that *N. caninum* is genetically unique within the same clade while being found in diverse geographical areas and hosts. Therefore, it seems that the ITS1 gene functions as an extremely sensitive marker for phylogenetic analysis as well as the diagnosis of neosporosis [19].

In this investigation, the prevalence of neosporosis was higher in cattle than in sheep and goats. Cattle’s increased vulnerability to neosporosis infection could be the reason for the disparity in prevalence rates between these animals [27]. Although natural infection in sheep and goats is uncommon [27,28], in our study, aborted fetuses from sheep and goats exhibited a significant positivity for *N. caninum*. Smallholder farmers’ practice of rearing these animals in a free-ranging system, which facilitates access by dogs, is responsible for this occurrence.

The significant risk factors for neosporosis found in this study are a time of abortion in pregnancy, the abortion history of the animals, the presence of the dog in the animal’s farm, and management practices. Other studies [20,29] also reported similar observations.

Researchers found *N. caninum *in the brain samples of aborted fetuses in many countries, including 5% in Brazil [30], 6.8% in Spain [31], 8.6% in Italy [32], and 18.9% in England [33]. Iran recorded 15.6% of aborted fetuses in sheep and no infection in goats [27]. The variation in the results can be due to variations in the management system as well as climate and environmental factors.

The study observed the highest prevalence of *N. caninum* in animals during the second trimester of pregnancy. According to the literature, most neosporosis-induced abortions occur at 4–6-month gestation [1,34], which supports the result of this study.

Evidence suggests that dogs on cattle farms are a risk factor for bovine neosporosis [7]. In the present study, dogs in rural areas had frequent and close contact with local breeds that graze freely on the pastures. Dogs are peridomestic, and they sometimes live with livestock. Therefore, this study and others [7,35] found that animals reared in a free-range system or on a farm with unrestricted access to dogs are more susceptible to *N. caninum* infections. We surmise that the higher prevalence of *N. caninum* infection in local breeds compared to crossbreeds might stem from their intimate interaction with dogs. This may increase the chances of abortion in local breeds [36].

In agreement with previous studies in Argentina, Venezuela, and Ethiopia [37,38], univariate analysis revealed that crossbreeds were less likely than local strains to acquire the infection. However, a previous study in Pakistan reported a higher infection rate in crossbred cattle compared to other cattle [39]. This variation could be due to differences in the systems for each breed’s production, as well as the disparity in infection susceptibility [35].

Irrespective of species, breed, and locality of animal, *N. caninum *was more prevalent (18.75%) in animals aged 2 to 4 years (Table 1). Similar observations were made by Metwally et al. [40], who reported 17% prevalence of *N. caninum* in the youngest age group (< 3 years) in Egypt and 19.1% in 1 to 3-year-old dairy cattle in south India [41].

Fertility is the most important factor affecting profitability in animal breeding [42]. In addition to ensuring timely animal reproduction, it is crucial that pregnancies do not result in abortions, as this can lead to economic losses in animal breeding [43]. Cumulatively, the findings of this study suggest an important role of *N. caninum* as a possible abortive agent for these animal species. Therefore, routine diagnosis is essential for investigating neosporosis in farm animals, particularly in herds experiencing pregnancy loss or interruptions. Moreover, we strongly advise implementing integrated control strategies and measures to combat neosporosis, given the prevalence of infection in the surveyed region of Bangladesh.

## Conclusion

Our study has unveiled *N. caninum* as a significant contributor to abortion in cattle, sheep, and goats. It is imperative to initiate awareness programs targeting both farmers and veterinarians to educate them about the risks associated with this parasite. Additionally, the development of prevention and control programs for neosporosis should consider the associated risk factors. To enhance our understanding of the molecular epidemiology of neosporosis, we strongly recommend further research involving larger sample sizes of other definitive and intermediate hosts.

## List of Abbreviations

°C, Degree Celcius; et al., And his associates; μl, Microliter; pmol, Picomole; BLAST, Basic Local Alignment Search Tool; DNA, Deoxyribonucleic Acid; IHC, Immunohistochemistry; ITS1, Internal Transcribed Spacer 1; nPCR, Nested polymerase chain reaction; PCR, Polymerase chain reaction; rDNA, Ribosomal DNA.
